# Social inequalities in injury occurrence and in disability retirement attributable to injuries: a 5 year follow-up study of a 2.1 million gainfully employed people

**DOI:** 10.1186/1471-2458-7-215

**Published:** 2007-08-23

**Authors:** Harald Hannerz, Kim L Mikkelsen, Martin L Nielsen, Finn Tüchsen, Søren Spangenberg

**Affiliations:** 1 National Institute of Occupational Health, Copenhagen, Denmark; 2 Department of Occupational Medicine, Hillerød Hospital, Hillerød, Denmark

## Abstract

**Background:**

Inequalities in injury related disability retirement may be due to differences in injury risk and or differences in retirement given injury. The aim of the present study was to measure social inequalities in injury occurrence and injury related disability retirement.

**Methods:**

All people in the Danish labour force aged 20–59 years 1 January 1997 were followed for injury related hospital contacts during 1997 and all people in the Danish labour force aged 21–54 years 1 January 1998 were followed for injury related hospital contacts during 1997 and for disability retirements during 1998–2002. As inequality indices we used excess fractions (EF) i.e. the proportions of the cases that would not have occurred if the risks in each social group had been as low as they were in the occupational group with the highest skill requirements.

**Results:**

With regard to the risk that an injury will occur, the EF was 36% among men and 10% among women. With regard to the risk that an injury will lead to disability retirement, the EF was 43% among men and 47% among women. The combined effect of the two types of inequalities rendered an EF for injury related disability retirement of 64% among men and 53% among women. The correlation between the case disability rate ratios among men and those among women was low (r = -0.110, P = 0.795).

**Conclusion:**

The social inequality in injury related disability retirement lies only to some degree in the differences in the injury risk. More important are differences in the consequences of an injury. This was especially pronounced among the women.

## Background

Several studies have dealt with social inequalities in disability retirement rates [1-5] and it is generally considered that social class is one of the most influential predictors of disability pension. The rates are typically higher among blue-collar workers than they are among white-collar workers. There is also a clear gradient among the white-collar workers, – the higher in the hierarchical class structure, the lower the rates [6].

A part of the inequalities in disability retirement rates would be due to inequalities in health and safety while the rest would be due to inequalities with regard to the risk of being excluded from the labour market, given acquired disability. The risks of disability are typically associated with the environment, genetic predisposition, acquired vulnerability, and risk behaviour while the exclusion risks among people with disabilities are associated with rehabilitation possibilities and factors that influence the inclusiveness of the labour market.

Studies on inequalities in health abound [7]. Similarly, the literature on return-to-work (RTW) [8,9], re-employment [9-13], and disability retirement [14] is extensive, but we have not found any studies that systematically estimate social inequalities in exclusion risks among disabled workers.

Disability retirement among worn-out manual workers has been regarded as a welcome compensation for a hard work-life. Recent research has, however, shown that 81% of all disability pensioners in Denmark retired involuntarily [15]. It has also been shown that involuntary labour market exclusion has adverse consequences on physical functioning, mental health and mortality even after baseline health and socio-demographic factors are controlled for [16,17]. In particular, it has been found that involuntary job loss is associated with increased risk for stroke [18], increased depressive symptoms [19] and increased risk of serious self-harm leading to hospital contacts or death [20].

Since primary and secondary prevention of disability retirement often require quite different types of prevention strategies it would be useful to differentiate between inequalities in health and safety and inequalities in exclusion probabilities among people with ill health and disabilities in the estimation of social inequalities in disability retirement. In particular we find it useful to estimate the contribution to the inequality in injury related disability retirement which is due to inequalities in injury risk at one hand and the inequality in disability retirement once injured on the other hand.

The aim of the present work was to measure occupational social status inequalities with regard to:

A. the risk that an injury will occur, and

B. the risk that an injury will lead to disability retirement.

Since injuries can lead to disability retirement regardless of cause and mechanism all injuries leading to hospital contacts are included, with no distinction between work and non-work related injuries.

## Methods

### Data source

The present study used information obtained through a record-linkage between three Danish national registers – the centralised civil registration system (CRS), the hospital patient register, and the employment classification module. The hospital patient register has existed since 1977 and contains data from all public hospitals in Denmark (more than 99% of all admissions). In the time period 1977–94, the register only included inpatients but from 1995 it also covers outpatients and emergency ward visits [21]. The diagnoses have been coded according to international classification of diseases version ten (ICD-10) since 1994. CRS contains information on gender, addresses and dates of birth, death and migrations for every person who is or has been an inhabitant of Denmark sometime between 1968 and present time. A person's employment status and occupation are registered annually in the employment classification module [21]. The occupations are coded in accordance with DISCO-88, which is the Danish version of the international standard classification of occupations (ISCO-88) [22]. In DISCO-88, the occupations are hierarchically divided into 10 major groups, 27 sub-major groups, 111 minor groups and 372 unit groups. The ten major groups are:

1. Legislators, senior officials and managers.

2. Professionals.

3. Technicians & associate professionals.

4. Clerks.

5. Service workers and shop and market sales workers.

6. Skilled agricultural and fishery workers.

7. Craft and related trades workers.

8. Plant and machinery operators and assemblers.

9. Elementary occupations.

0. Armed forces.

The major groups 0 and 1 (which together comprise approximately seven percent of all economically active people in Denmark) are considered heterogeneous with respect to social status and will for that reason not be included in the calculations of social inequalities. The other major groups will serve as proxies for social status groups, with group 2 "professionals" as the reference group. The study was conducted in accordance with the ethical rules and regulation of, and was approved by, the Danish Data Inspection service.

### Calculations with regard to the risk that an injury will occur

All economically active persons in Denmark aged 20–59 years 1 January 1997 were followed in the hospital register of 1997 for the first occurrence of a hospital contact with a principal diagnosis in the ICD-10 interval S00-T98 (injury, poisoning and certain other consequences of external causes). Subjects were followed to the end of the follow-up period unless the sought outcome occurred or they died or emigrated, in which case the follow-up ended since the subjects were no longer at risk.

For each gender, we used indirect standardisation to adjust for age in five-year age groups, with all economically active people in the total population of Denmark as standard population, and we estimated standardised incidence ratios by social group.

The excess fraction, i.e. the proportion of the cases that would not have happened if the injury incidence rate in every social group had been as low as that in the reference group, was estimated by the equation

EFINCIDENCE=∑ipi(IRRi−1)1+∑ipi(IRRi−1)
 MathType@MTEF@5@5@+=feaafiart1ev1aaatCvAUfKttLearuWrP9MDH5MBPbIqV92AaeXatLxBI9gBaebbnrfifHhDYfgasaacH8akY=wiFfYdH8Gipec8Eeeu0xXdbba9frFj0=OqFfea0dXdd9vqai=hGuQ8kuc9pgc9s8qqaq=dirpe0xb9q8qiLsFr0=vr0=vr0dc8meaabaqaciaacaGaaeqabaqabeGadaaakeaacqWGfbqrcqWGgbGrdaWgaaWcbaGaemysaKKaemOta4Kaem4qamKaemysaKKaemiraqKaemyrauKaemOta4Kaem4qamKaemyraueabeaakiabg2da9maalaaabaWaaabuaeaacqWGWbaCdaWgaaWcbaGaemyAaKgabeaaaeaacqWGPbqAaeqaniabggHiLdGcdaqadaqaaiabdMeajjabdkfasjabdkfasnaaBaaaleaacqWGPbqAaeqaaOGaeyOeI0IaeGymaedacaGLOaGaayzkaaaabaGaeGymaeJaey4kaSYaaabuaeaacqWGWbaCdaWgaaWcbaGaemyAaKgabeaaaeaacqWGPbqAaeqaniabggHiLdGcdaqadaqaaiabdMeajjabdkfasjabdkfasnaaBaaaleaacqWGPbqAaeqaaOGaeyOeI0IaeGymaedacaGLOaGaayzkaaaaaaaa@5979@

where *p*_*i *_is the proportion of the study population that belong to group *i*, and *IRR*_*i *_is the standardised incidence ratio in social group *i *divided by that in group 2 "Professionals", which was the predetermined reference group.

### Calculations with regard to the risk that an injury will lead to disability retirement

All economically active persons in Denmark aged 21–54 years 1 January 1998 were followed in the period 1998–2002 for the first occurrence of the socio-economic status 'disability pensioner' in the employment classification module. If a disability retirement occurred during a certain calendar year, the retirement date was set to July 1^st ^that year. Subjects were followed to the end of the follow-up period unless the sought outcome occurred or they died, emigrated or retired for other reasons than disability, in which case the follow-up ended since the subjects were no longer at risk. Record-linkage to the hospital register was done for information on hospital treatment for an injury (ICD-10: S00-T98) during 1997 – the year preceding the baseline date. For each gender, we used indirect standardisation to adjust for age in five-year age groups, with all economically active people in the total population of Denmark as standard population, and we estimated standardised incidence ratios for disability retirement by social group and injury status, defined by whether or not the person was treated for an injury during 1997.

For each social group, we then estimated the proportion of the observed disability retirements among the people with an injury at baseline that could be attributed to the baseline injury. For this we used the equation

APi=SDRi,Injured−SDRi,Non−injuredSDRi,Injured
 MathType@MTEF@5@5@+=feaafiart1ev1aaatCvAUfKttLearuWrP9MDH5MBPbIqV92AaeXatLxBI9gBaebbnrfifHhDYfgasaacH8akY=wiFfYdH8Gipec8Eeeu0xXdbba9frFj0=OqFfea0dXdd9vqai=hGuQ8kuc9pgc9s8qqaq=dirpe0xb9q8qiLsFr0=vr0=vr0dc8meaabaqaciaacaGaaeqabaqabeGadaaakeaacqWGbbqqcqWGqbaudaWgaaWcbaGaemyAaKgabeaakiabg2da9maalaaabaGaem4uamLaemiraqKaemOuai1aaSbaaSqaaiabdMgaPjabcYcaSiabdMeajjabd6gaUjabdQgaQjabdwha1jabdkhaYjabdwgaLjabdsgaKbqabaGccqGHsislcqWGtbWucqWGebarcqWGsbGudaWgaaWcbaGaemyAaKMaeiilaWIaemOta4Kaem4Ba8MaemOBa4MaeyOeI0IaemyAaKMaemOBa4MaemOAaOMaemyDauNaemOCaiNaemyzauMaemizaqgabeaaaOqaaiabdofatjabdseaejabdkfasnaaBaaaleaacqWGPbqAcqGGSaalcqWGjbqscqWGUbGBcqWGQbGAcqWG1bqDcqWGYbGCcqWGLbqzcqWGKbazaeqaaaaaaaa@6545@

where *AP*_*i *_is the attributable proportion in social group *i *and *SDR*_*i*,*Injured *_is the standardised disability ratio (standardised incidence ratio for disability retirement) among the people in group *i *who were injured at baseline.

The relative risk for an injury related disability retirement among the injured was estimated by the equation

CDRRi=APi×SDRi,InjuredAP2×SDR2,Injured
 MathType@MTEF@5@5@+=feaafiart1ev1aaatCvAUfKttLearuWrP9MDH5MBPbIqV92AaeXatLxBI9gBaebbnrfifHhDYfgasaacH8akY=wiFfYdH8Gipec8Eeeu0xXdbba9frFj0=OqFfea0dXdd9vqai=hGuQ8kuc9pgc9s8qqaq=dirpe0xb9q8qiLsFr0=vr0=vr0dc8meaabaqaciaacaGaaeqabaqabeGadaaakeaacqWGdbWqcqWGebarcqWGsbGucqWGsbGudaWgaaWcbaGaemyAaKgabeaakiabg2da9maalaaabaGaemyqaeKaemiuaa1aaSbaaSqaaiabdMgaPbqabaGccqGHxdaTcqWGtbWucqWGebarcqWGsbGudaWgaaWcbaGaemyAaKMaeiilaWIaemysaKKaemOBa4MaemOAaOMaemyDauNaemOCaiNaemyzauMaemizaqgabeaaaOqaaiabdgeabjabdcfaqnaaBaaaleaacqaIYaGmaeqaaOGaey41aqRaem4uamLaemiraqKaemOuai1aaSbaaSqaaiabikdaYiabcYcaSiabdMeajjabd6gaUjabdQgaQjabdwha1jabdkhaYjabdwgaLjabdsgaKbqabaaaaaaa@5D27@

where the case disability rate ratio (*CDRR*_*i*_) gives the ratio of the risk that an injury will lead to disability retirement in social group *i *with that in social group 2 "Professionals", which was the predetermined reference group.

Propagation of error formulas [23] and large sample theory [24] were used to form confidence intervals around the estimates.

The excess fraction, i.e. the proportion of the injury related disability retirements that would not have happened if the injury related case disability rate in every social group had been as low as that in the reference group, was estimated by the equation

EFCASE_DISABILITY=∑iqi(CDRRi−1)1+∑iqi(CDRRi−1)e
 MathType@MTEF@5@5@+=feaafiart1ev1aaatCvAUfKttLearuWrP9MDH5MBPbIqV92AaeXatLxBI9gBaebbnrfifHhDYfgasaacH8akY=wiFfYdH8Gipec8Eeeu0xXdbba9frFj0=OqFfea0dXdd9vqai=hGuQ8kuc9pgc9s8qqaq=dirpe0xb9q8qiLsFr0=vr0=vr0dc8meaabaqaciaacaGaaeqabaqabeGadaaakeaacqWGfbqrcqWGgbGrdaWgaaWcbaGaem4qamKaemyqaeKaem4uamLaemyrauKaei4xa8LaemiraqKaemysaKKaem4uamLaemyqaeKaemOqaiKaemysaKKaemitaWKaemysaKKaemivaqLaemywaKfabeaakiabg2da9maalaaabaWaaabuaeaacqWGXbqCdaWgaaWcbaGaemyAaKgabeaaaeaacqWGPbqAaeqaniabggHiLdGcdaqadaqaaiabdoeadjabdseaejabdkfasjabdkfasnaaBaaaleaacqWGPbqAaeqaaOGaeyOeI0IaeGymaedacaGLOaGaayzkaaaabaGaeGymaeJaey4kaSYaaabuaeaacqWGXbqCdaWgaaWcbaGaemyAaKgabeaaaeaacqWGPbqAaeqaniabggHiLdGcdaqadaqaaiabdoeadjabdseaejabdkfasjabdkfasnaaBaaaleaacqWGPbqAaeqaaOGaeyOeI0IaeGymaedacaGLOaGaayzkaaaaaiabdwgaLbaa@63DD@

where *q*_*i *_is the proportion of the injury cases that belong to group *i*.

### The combined effect of inequalities in injury incidence rates and case disability rates

The excess fraction, i.e. the proportion of the injury related disability retirements that would not have happened if the injury related disability rate in every social group had been as low as that in the reference group, was estimated by the equation

EFDISABILITY=∑ipi(IRRi×CDRRi−1)1+∑ipi(IRRi×CDRRi−1)
 MathType@MTEF@5@5@+=feaafiart1ev1aaatCvAUfKttLearuWrP9MDH5MBPbIqV92AaeXatLxBI9gBaebbnrfifHhDYfgasaacH8akY=wiFfYdH8Gipec8Eeeu0xXdbba9frFj0=OqFfea0dXdd9vqai=hGuQ8kuc9pgc9s8qqaq=dirpe0xb9q8qiLsFr0=vr0=vr0dc8meaabaqaciaacaGaaeqabaqabeGadaaakeaacqWGfbqrcqWGgbGrdaWgaaWcbaGaemiraqKaemysaKKaem4uamLaemyqaeKaemOqaiKaemysaKKaemitaWKaemysaKKaemivaqLaemywaKfabeaakiabg2da9maalaaabaWaaabuaeaacqWGWbaCdaWgaaWcbaGaemyAaKgabeaaaeaacqWGPbqAaeqaniabggHiLdGcdaqadaqaaiabdMeajjabdkfasjabdkfasnaaBaaaleaacqWGPbqAaeqaaOGaey41aqRaem4qamKaemiraqKaemOuaiLaemOuai1aaSbaaSqaaiabdMgaPbqabaGccqGHsislcqaIXaqmaiaawIcacaGLPaaaaeaacqaIXaqmcqGHRaWkdaaeqbqaaiabdchaWnaaBaaaleaacqWGPbqAaeqaaaqaaiabdMgaPbqab0GaeyyeIuoakmaabmaabaGaemysaKKaemOuaiLaemOuai1aaSbaaSqaaiabdMgaPbqabaGccqGHxdaTcqWGdbWqcqWGebarcqWGsbGucqWGsbGudaWgaaWcbaGaemyAaKgabeaakiabgkHiTiabigdaXaGaayjkaiaawMcaaaaaaaa@6B1E@

where *p*_*i *_is the proportion of the study population that belong to group *i*.

## Results

The part of the study that dealt with social inequalities in injury incidence during 1997 included 1 101 714 men and 1 034 903 women (the total number of people and the number of injured people in each social group is given in table 1). 87% of the emergency ward visits were accidents while 2% were intentional. Nine percent of the accidents were traffic-related and 27% were fall-related. Among the people who were economically active 1 January 1998, 139 235 men and 78 411 women were injured in 1997. In total, we observed 2257 disability retirements among the male cases and 1906 among the female cases. According to equation (2), 37% of the retirements among the male cases and 44% of the retirements among the female cases could be statistically attributed to an injury at baseline.

**Table 1:** Number of men and women in the study population 1997, by social group

		Men		Women	
		Total	Injured	Total	Injured
2	Professionals	168504	12515	122556	8059
3	Technicians and associate professional	155793	14217	229480	17170
4	Clerks	78235	9025	237654	16900
5	Service workers and shop and market sales workers	82580	12108	253768	21510
6	Skilled agricultural and fishery workers	46974	4331	5649	437
7	Craft and related trades workers	273230	43462	20591	1969
8	Plant and machine operators and assemblers	151612	22604	55458	4998
9	Elementary occupations	144786	22910	109747	9181

With regard to social inequalities in the risk that an injury will occur, we observed an excess fraction (*EF*_INCIDENCE_) of 36% among the men and 10% among the women. With regard to social inequalities in the risk that an injury will lead to disability retirement, we observed an excess fraction (*EF*_CASE_DISABILITY_) of 43% among the men and 47% among the women. Finally, the combined effect of inequalities in injury incidence rates and case disability rates gave us an excess fraction for injury related disability retirement (*EF*_DISABILITY_) of 64% among the men and 53% among the women.

### Rate ratios by social group are given in table 2, 3

**Table 2:** Rate ratios, by social group, with 95% confidence intervals (CI) for men

Social group		Injury incidence rate ratio	95% CI	Injury related case disability retirement rate ratio	95% CI	Injury related disability retirement rate ratio	95% CI
2	Professionals	1.00	--	1.00	--	1.00	--
3	Technicians and associate professional	1.17	(1.16–1.19)	1.34	(0.99–1.81)	1.58	(1.17–2.13)
4	Clerks	1.25	(1.23–1.28)	1.75	(1.20–2.56)	2.20	(1.50–3.21)
5	Service workers and shop and market sales workers	1.55	(1.52–1.58)	1.67	(1.19–2.34)	2.59	(1.85–3.64)
6	Skilled agricultural and fishery workers	1.25	(1.21–1.29)	0.95	(0.55–1.64)	1.19	(0.69–2.05)
7	Craft and related trades workers	1.92	(1.90–1.94)	1.48	(1.16–1.88)	2.84	(2.24–3.61)
8	Plant and machine operators and assemblers	1.84	(1.82–1.87)	2.21	(1.72–2.84)	4.07	(3.17–5.23)
9	Elementary occupations	1.89	(1.86–1.91)	2.72	(2.14–3.46)	5.12	(4.02–6.52)

**Table 3:** Rate ratios, by social group, with 95% confidence intervals (CI) for women

Social group		Injury incidence rate ratio	95% CI	Injury related case disability retirement rate ratio	95% CI	Injury related disability retirement rate ratio	95% CI
2	Professionals	1.00	--	1.00	--	1.00	--
3	Technicians and associate professional	1.12	(1.10–1.13)	1.49	(1.19–1.86)	1.66	(1.33–2.08)
4	Clerks	1.00	(0.99–1.02)	1.60	(1.24–2.06)	1.60	(1.24–2.07)
5	Service workers and shop and market sales workers	1.17	(1.16–1.19)	2.28	(1.81–2.87)	2.68	(2.13–3.38)
6	Skilled agricultural and fishery workers	1.11	(1.01–1.22)	5.20	(2.77–9.75)	5.77	(3.06–10.90)
7	Craft and related trades workers	1.30	(1.25–1.36)	2.40	(1.47–3.94)	3.13	(1.91–5.14)
8	Plant and machine operators and assemblers	1.29	(1.25–1.33)	1.78	(1.25–2.52)	2.29	(1.62–3.26)
9	Elementary occupations	1.21	(1.19–1.24)	2.96	(2.30–3.80)	3.58	(2.78–4.61)

The possible causes underlying the social inequality in disability retirement due to an injury might be segregated into two sets of factors. One set of factors causing the injury, and one set of factors causing the retirement given the injury. The set of rate ratios across the social groups, for example for injuries, might be seen as a reflection of the set of underlying causal factors. Therefore the correlation between these sets of rate ratios may be seen as a crude measure of how much the sets of underlying causal factors overlap. For men there seems to be an extensive overlap between the set of causes leading to an injury and the set of causes leading to disability retirement given an injury, as the correlation between the injury rate ratios and the case disability rate ratios across the social groups is high (r = 0.730, P = 0.040). In females on the other hand, these two sets of causes are presumably different as the correlation here is low (r = 0.179, P = 0.671). The set of factors causing injuries is probably similar for men and women as the correlation between the injury rate ratios for men and women is very high (r = 0.907, P = 0.002). Corresponding to this, the set of factors causing an injured person to retire are presumably different for men and women as the correlation between the case disability rate ratios is low (r = -0.110, P = 0.795).

## Discussion

We found large inequalities in injury related disability retirement. The excess fractions indicate that 64% of the concerned disability retirements among the men and 53% among the women would not have occurred if the risks in each socio-occupational group had been as low as they were among professionals (the reference group). We also found that the majority of the inequalities was due to differences in the case disability rate (the risk that an injury would lead to disability retirement). This was especially pronounced among the women, where the impact of differences in case disability rates was nearly five times greater than that of differences in injury incidence. A high contribution to the inequality due to differences in the case disability rate relative to the difference in injury risk was seen for the social group 'skilled agricultural and fishery workers'. In this group low injury rate and high case disability retirement rate is however consistent with low self-referral for hospital treatment, which might be a phenomenon among skilled agricultural and fishery workers. Another important finding was the lack of correlation between the case disability ratios among women and those among men.

The prospective design strengthens the study and we believe that the chosen follow-up period (5 years) was long enough to capture the vast majority of all disability retirements before age 60, which were or will be caused by an injury at baseline. Another strength was that the calculation of the attributable proportions was done through a statistical model (Equation 2), which is independent of retrospective medical opinions and legal considerations with regard to what cause should be assigned to a particular disability retirement. In Denmark, a person is eligible for early retirement at age 60. Therefore, a person can, but do not need to, seek disability retirement if he or she incurs a disability after 60. We cannot know to what extent the early retirements (age 60 and over) are due to disability. But we know that we would have a problem with false negative cases if we were to study disability retirements in this age group. For this reason we chose to only follow people until age 60.

In countries with a mix of private and public hospitals, referral bias is a major methodological problem [25]. The Danish referral system is unified and private hospitals are very few and deal with non-acute treatment. For admissions due to injuries geographical distances are important. Denmark has fully-funded public medical care and hospital system. Therefore neither accessibility nor economic incentives are likely to cause different treatment rates for various social strata. It has been shown, however, that on-site medical facilities have a potential for reducing the use of acute ward treatment [26]. That is especially true for medical facilities manned with professional medical staff. Such facilities are, however, very few in Denmark so at the most they may have reduced the social inequality in hospital treatments slightly. The counties own the hospitals in Denmark and the counties have very different policies for access to emergency wards causing a significant geographical difference in the ratios of treatments by GPs and emergency wards [21]. We have not adjusted for this referral bias. A similar bias by county may exist because a board in each local community decides the pension entitlement. A national complaints board shall, however, ensure that all citizens are treated equal.

We analysed the effect of injuries observed in 1997 on disability retirement in the following five years 1998–2002. The study design has some implications for the inference of the results. Using excess fraction as our measure of inequality, we do not look at disability retirements classified as caused by a specific injury in individuals, instead we look at the amount of all disability retirements in populations that can be attributed to the injuries observed at baseline. Attributable proportions can be calculated without knowing the actual causes for specific individuals. Furthermore, the relative rates estimated and the derived attributable proportions and excess fractions are not the effects of the counterfactual 'having an injury vs. not having an injury', but the effects of the counterfactual 'having an injury in 1997 vs. not having an injury in 1997'. Therefore, it is not possible to know whether the specific injury observed in 1997 actually caused the subsequent disability retirement for that specific individual. Rather, the increased rate of disability retirement in the injured group must be understood as an effect of the injuries, with the injury as the main cause, a contributing cause or a trigger, or the injury is merely associated with other incidences of injuries or with other causes of disability.

The main groups of DISCO-88 were used as a proxy measure of social status, and professionals were predefined as the group with the highest social status. It must be assumed that there is no intra-group heterogeneity. To the extent that a gradient of social status exist within the main groups of DISCO-88, specifically within the reference group of professionals, our excess fraction estimates will be conservative.

Injury related hospital contacts are strongly associated with social status. The three main sources of injury related hospital contacts are work, traffic and sports. The last mentioned source does not have the same social gradient as the other two. Work related injuries leading to hospitalization are much more common in manual industries like slaughterhouses, manufacture of wood and wood products than in administrative and clerical work and among professionals [27].

The gender differences in our results are significant in showing that the impact of differences in the consequences of an injury relative to the impact of differences in injury risk is stronger in women than in men. Further our results indicate that the factors contributing to disability retirement once injured are different for men and women. However, we have no data on what these different factors might be. However, as the relative rates of having an injury seem to follow the natural order of the occupational social status groups, the set of factors leading to injuries, in both men and women, and the set of factors in men leading to disability retirement once injured, is at least to some extent work related. The causes leading to retirement once injured in women might differ from those in men and the causes may to a lesser extent be work related in women.

Many different factors at societal, organisational, occupational, and individual levels are likely to contribute to the inequality between occupations in the risk of ending up on disability pension if exposed to an injury. If health care is of insufficient quality or unaffordable for parts of the population, some injury victims – typically of lower socio-economic status – will have more lasting sequelae than with optimal treatment, and thus a higher risk of disability pension. Legal and economical incentives and sanctions can strongly influence the possibilities of being employed if partly disabled after an injury. Rules against discrimination, support for education, technical or personal aides, incentives for the employer and many other circumstances can be crucial in this respect.

The policies and practices of the organisation and the occupation of the injured worker will also have a large impact on further employment. In a situation with incomplete recovery after the injury, work tasks will often determine the level of symptoms in daily life. Organisations and occupations that allow for a diverse choice of tasks will provide better possibilities to place the injured victim in a job that do not provoke unbearable pain or other symptoms, which may cause the worker to opt for disability retirement.

As workers with lower skills are usually considered more easily replaced than higher skilled employees, only deliberate efforts to adapt work to the injured workers abilities, regardless of occupational status, can counteract a tendency of unequal exclusion. Many initiatives have been taken during the last decade by the European Community in order to ensure people with disabilities employment in ordinary jobs, e.g. personal assistance, preference to certain public jobs, financial support to adaptations in the workplace and to acquisition of special tools, but a Danish study [28] shows that the majority of the Danish companies make no use of these initiatives because of attitudinal barriers among management and employees and a lack of knowledge about the public financial support.

A recent study about return to work among disabled workers in the US [32] underlines the importance of job analysis, and of educating other workers about disability.

Factors like skills, motivation, flexibility, union membership, and many others, are likely to influence the possibilities to change job tasks – within or outside the usual occupation and workplace – if the injury consequences made it impossible to carry on with the usual tasks. As at least education and skills are very unevenly distributed between occupations, these factors will also contribute to the inequality in disability. Part of the absence from work must be considered due to individual coping with work demands [29].

A Danish record linkage study has shown that although waiting time for hospital examination and treatment were similar for different occupational groups with musculoskeletal problems, the unskilled workers were on sick leave much more of the waiting time than professionals [30]. We consider the differences in work demands and thereby symptoms to be the most likely explanation for this difference.

A Canadian group has shown that health care interventions have limited impact on return to work for long-term sick-listed workers, whereas workplace interventions including ergonomic improvements and modified work were more efficient, and a combined intervention had the most profound impact [31]. Although this intervention was more costly, it produced the largest overall savings due to a pronounced reduction in compensation of lost income over the following 5 years. Ergonomic interventions also proved effective in a six-country study [32]. In the US study of work incapacity and reintegration, 80 percent of respondents who resumed working did so with the help of workplace accommodations [33].

## Conclusion

The social inequality in injury related disability retirement lies only to some degree in the differences in the injury risk. More important are differences in the consequences of an injury. This was especially pronounced among the women, where the impact of differences in the consequences of an injury was nearly five times greater than the impact of differences in injury risk. For men, the impact of the inequality in injury risk and the impact of the inequality in the consequences of an injury were highly correlated, whereas this was not the case for women.

## Competing interests

The author(s) declare that they have no competing interests.

## Authors' contributions

All authors have made substantial contributions to conception and design and have been involved in drafting the manuscript. HH performed the statistical analysis. HH, KLM and FT have critically revised the manuscript. All authors have read and approved the final manuscript.

## Pre-publication history

The pre-publication history for this paper can be accessed here:


